# 2-Methoxyethanol: MAK-Begründung, Nachtrag

**DOI:** 10.34865/mb10986d10_1ad

**Published:** 2025-03-31

**Authors:** Andrea Hartwig, MAK Commission

**Affiliations:** 1 Institut für Angewandte Biowissenschaften. Abteilung Lebensmittelchemie und Toxikologie. Karlsruher Institut für Technologie (KIT) Adenauerring 20a, Geb. 50.41 76131 Karlsruhe Deutschland; 2 Ständige Senatskommission zur Prüfung gesundheitsschädlicher Arbeitsstoffe. Deutsche Forschungsgemeinschaft, Kennedyallee 40, 53175 Bonn, Deutschland. Weitere Informationen: Ständige Senatskommission zur Prüfung gesundheitsschädlicher Arbeitsstoffe | DFG

**Keywords:** 2-Methoxyethanol, Entwicklungstoxizität, Fertilität, Hämatotoxizität, MAK-Wert, maximale Arbeitsplatzkonzentration, Schwangerschaftsgruppe

## Abstract

The German Senate Commission for the Investigation of Health Hazards of Chemical Compounds in the Work Area (MAK Commission) re-evaluated the data for developmental toxicity and the derivation of the occupational exposure limit value (maximum concentration at the workplace, MAK value) for 2-methoxyethanol [109-86-4]. The MAK value was derived in analogy to the BAT value (biological tolerance value) for the metabolite methoxyacetic acid of 15 mg methoxyacetic acid/g creatinine. This concentration was extrapolated to a 2-methoxyethanol concentration in air using the findings of a volunteer study and toxicokinetic considerations. The MAK value of 1 ml/m^3^ was retained based on these results. The substance remains classified in Pregnancy Risk Group B because a risk for the developing foetus cannot be ruled out at exposure to the MAK value of 1 ml/m^3^. This was concluded based on embryo-/foetotoxic and teratogenic effects induced by the metabolite methoxyacetic acid in rats, mice, rabbits and monkeys. A 2-methoxyethanol concentration of 0.15 ml/m^3^ was derived for the workplace from the lowest NOAEC (no observed adverse effect concentration) of 3 ml/m^3^ for developmental toxicity induced in rabbits by 2-methoxyethanol; damage to the embryo or foetus is unlikely at this concentration (the prerequisite for assignment to Pregnancy Risk Group C).

**Table:** 

**MAK-Wert (2009)**	**1 ml/m^3^ (ppm) ≙ 3,2 mg/m^3^^a)^**
**Spitzenbegrenzung (2001)**	**Kategorie II, Überschreitungsfaktor 8**

**Hautresorption (1980)**	**H**
**Sensibilisierende Wirkung**	**–**
**Krebserzeugende Wirkung**	**–**
**Fruchtschädigende Wirkung (1985)**	**Gruppe B^b)^**
**Keimzellmutagene Wirkung**	**–**

**BAT-Wert (2008)**	**15 mg Methoxyessigsäure/g Kreatinin**

CAS-Nr.	109-86-4
**1 ml/m^3^ (ppm) ≙ 3,16 mg/m^3^**	**1 mg/m^3^ ≙ 0,317 ml/m^3^ (ppm)**

^a)^ MAK-Wert für die Summe der Luftkonzentrationen von 2-Methoxyethanol und 2-Methoxyethylacetat

^b)^ Hinweis auf Voraussetzung für Gruppe C siehe Abschnitt 6

Im Jahr 2023 wurde die Zuordnung zu einer Schwangerschaftsgruppe für den BAT-Wert von 15 mg Methoxyessigsäure/g Kreatinin evaluiert. Dabei wurde auch ein Hinweis auf Voraussetzung für Gruppe C in Höhe von 2,5 mg Methoxyessigsäure/g Kreatinin vergeben (Michaelsen et al. 2024).

Seit dem letzten Nachtrag zur Begründung des MAK-Werts (Hartwig 2009) sind neue Studien hinzugekommen, die ergänzt werden. In diesem Nachtrag wird der MAK-Wert reevaluiert und geprüft, ob ein Hinweis auf Voraussetzung für Gruppe C gegeben werden kann.

## 1 Allgemeiner Wirkungscharakter

Für die toxischen Wirkungen von 2-Methoxyethanol ist der Hauptmetabolit Methoxyessigsäure verantwortlich.

Zielorgane von 2-Methoxyethanol sind beim Tier nach wiederholter Exposition unabhängig vom Aufnahmeweg die blutbildenden und lymphatischen Organe, das Blut, die Hoden und in geringerem Ausmaß Nieren, Leber, Nervensystem und Ovarien. Beim Menschen steht die Veränderung hämatologischer Parameter im Vordergrund.

Unverdünntes 2-Methoxyethanol ist bei Kaninchen weder haut- noch augenreizend.

Es liegen keine Hinweise auf eine sensibilisierende Wirkung vor.

2-Methoxyethanol ist entwicklungstoxisch und führt zu Fehlbildungen am Skelett und an inneren Organen. In pränatalen Entwicklungstoxizitätsstudien kommt es bei Ratten ab 25 ml 2-Methoxyethanol/m^3^, bei Mäusen ab 50 ml 2-Methoxyethanol/m^3^ und bei Kaninchen ab 10 ml 2-Methoxyethanol/m^3^ zu entwicklungstoxischen Effekten.

In vitro induziert 2-Methoxyethanol bei hohen Konzentrationen, ab 65 mM klastogene Effekte, die in vivo nicht bestätigt worden sind.

Untersuchungen zur Kanzerogenität liegen nicht vor.

## 2 Wirkungsmechanismus

2-Methoxyethanol führt bei Ratten, Mäusen, Kaninchen und Affen zu Schäden an den Hoden und Spermien. Für diese Effekte werden bei Ratten und Affen Apoptose und oxidativer Stress verantwortlich gemacht (Adeyemo-Salami und Farombi 2018; Sakurai et al. 2015; Starek-Świechowicz et al. 2015). In einer Metabolomanalyse von Serum, Urin, Leber und Hoden zeigte sich bei männlichen mit 2-Methoxyethanol behandelten Ratten, dass die Inhibierung der Cholinoxidation, des Katabolismus von verzweigtkettigen Aminosäuren sowie der Fettsäure-β-oxidation als erste bedeutsame metabolische Störungen auftreten. Diese zogen eine Akkumulation von Sarkosin, Dimethylglycin und verschiedener Carnitin- und Glycin-konjugierter Metaboliten nach sich. Die Autoren postulierten, dass die Inhibierung der Flavoproteindehydrogenasen-katalysierten Reaktionen den Wirkungsmechanismus für die 2-Methoxyethanol-induzierte Toxizität darstellt (Takei et al. 2010). An TM3-Leydig-Zellen von Mäusen ist gezeigt worden, dass der Hauptmetabolit Methoxyessigsäure die transkriptionelle Aktivität des Androgenrezeptors indirekt über den Tyrosinkinase-Signalweg, der die PI3-Kinase (Phosphoinositid-3-Kinase) beinhaltet, moduliert (Bagchi et al. 2009).

2-Methoxyethanol hat bei weiblichen Ratten über Methoxyessigsäure eine Hyperplasie der Corpora lutea zur Folge, was über eine Hochregulierung der Expression steroidogener Faktoren und einer Herunterregulierung der Expression luteolytischer Faktoren geschieht. Dies resultiert in einer Zunahme der Progesteronsekretion der neu gebildeten Corpora lutea im aktuellen Zyklus über direkte und indirekte Prolaktin-unabhängige Wege. Zudem stimuliert 2-Methoxyethanol direkt die Expression von 3β-HSD (3-β-Hydroxysteroid-Dehydrogenase) in alten Corpora lutea, die von vorhergehenden Zyklen verblieben sind (Taketa et al. 2011 b). Für Methoxyessigsäure wird angenommen, dass der Effekt auf die weibliche Reproduktion wenigstens zum Teil auf der Abschwächung des endogenen Östrogenrezeptor-vermittelten Signalwegs beruht (Henley et al. 2009).

Im Addendum zur Begründung des BAT-Werts (Michaelsen et al. 2024) ist dargestellt, dass die entwicklungstoxischen/teratogenen Effekte von 2-Methoxyethanol mit der Beeinflussung der De-novo-Biosynthese von Purinen, der Inhibierung der DNA-Synthese und mit Veränderungen der Acetylierungsprogrammierung von Histonen in Zusammenhang stehen.

## 3 Toxikokinetik und Metabolismus

Hierzu liegen keine neuen Studien vor.

Im Folgenden werden die für die Bewertung relevanten Studien nochmals beschrieben.

### 3.1 Toxikokinetik

#### 3.1.1 Mensch

Sieben männliche Probanden wurden mittels einer Atemmaske über Nase und Mund in Ruhe viermal je 50 Minuten gegen 16 mg 2-Methoxyethanol/m^3^ (5 ml/m^3^) exponiert. Nach jeder Expositionsphase folgte eine Pause mit einer Dauer von zehn Minuten, die gesamte Expositionszeit betrug 200 Minuten. Vor der Exposition sowie stündlich bis zum Ende der Exposition und im Verlauf der folgenden fünf Tage wurden Urinproben genommen. Alle Proben wurden gaschromatographisch auf Methoxyessigsäure untersucht. Mittels der Bestimmung der Atemvolumina lässt sich eine mittlere Gesamtaufnahme von 19,4 mg 2-Methoxyethanol innerhalb der 200 Minuten errechnen. Für Methoxyessigsäure wurde eine Eliminationshalbwertszeit von 77 Stunden angegeben (mittlere Gesamt-Wiederfindung im Urin nach 24 h: 15,3 % (Groeseneken et al. 1989).

**Abb. 1:**
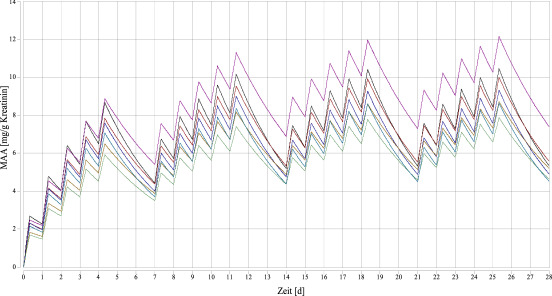
Simulation der Konzentrationen von Methoxyessigsäure im Urin nach inhalativer Exposition gegen 1 ml 2-Methoxyethanol/m^3^ basierend auf den Daten der Probandenstudie von Groeseneken et al. 1989 (SCOEL 2006)

Basierend auf den Daten der Studie von Groeseneken et al. (1989) wurde eine Simulation für die 4-wöchige Entwicklung der Urinkonzentrationen von Methoxyessigsäure nach inhalativer Exposition gegen 1 ml 2-Methoxyethanol/m^3^ vorgenommen. Dazu wurde ausgegangen von einer Exposition am Arbeitsplatz von fünf Tagen in der Woche und acht Stunden pro Tag. In der Simulation wurde ein Ein-Kompartiment-Modell eingesetzt und eine zeitunabhängige (konstante Geschwindigkeiten, keine Enzyminduktion) lineare (keine metabolische Sättigung) Kinetik angenommen, d. h. die Halbwertszeit ist unabhängig von der Konzentration (SCOEL 2006). Aus der Abbildung im Appendix des Berichtes von SCOEL lässt sich entnehmen, dass die Ausscheidung von Methoxyessigsäure innerhalb der 5-Tage-Woche zunimmt (am 5. Tag nach acht Stunden ca. 3-fache Menge wie am Ende des ersten Expositionstages). Nach ca. vier Wochen hat sich ein Fließgleichgewicht eingestellt. Dann beträgt am fünften Tag der Woche die Ausscheidung von Methoxyessigsäure ca. das 5-Fache im Vergleich zum ersten Expositionstag („Akkumulationsfaktor“ etwa 5) (siehe Abbildung 1).

MAA: Methoxyessigsäure

Für den Menschen ist eine hohe dermale Aufnahme von 2-Methoxyethanol aus der Gasphase nachgewiesen. Bei Ganzkörper-Dampfexposition liegt die dermale Aufnahme bei 55 % und die respiratorische Aufnahme bei 45 % (Kežić et al. 1997).

**Abb. 2:**
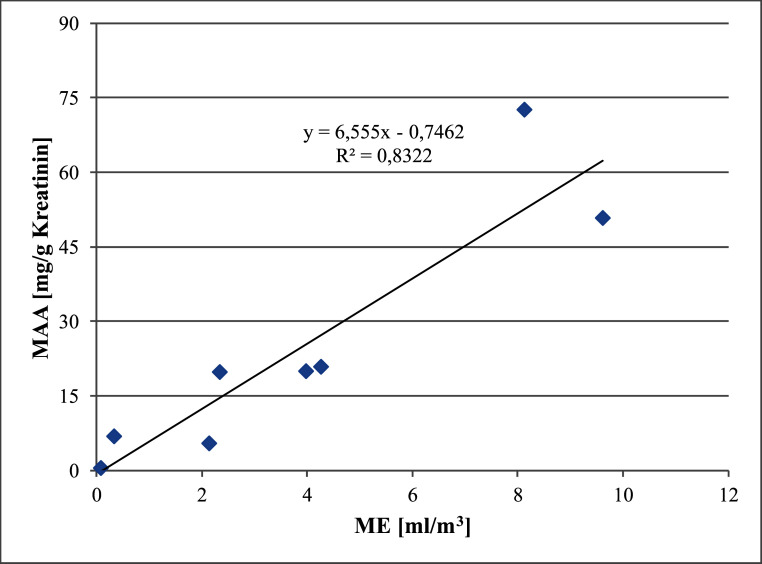
Korrelation zwischen der Konzentration von 2-Methoxyethanol in der Luft und der Konzentration von Methoxyessigsäure im Urin (geometrische Mittelwerte) aus arbeitsmedizinischen Studien (Chang et al. 2004; Shih et al. 2000, 2003)

Im Nachtrag von 2009 (Hartwig 2009) wurde eine Korrelation der Luftkonzentration von 2-Methoxyethanol und der Urinkonzentration des Metaboliten Methoxyessigsäure aus arbeitsmedizinischen Studien dargestellt. Im vorliegenden Nachtrag wird eine Korrektur der zugrundeliegenden Tabelle vorgenommen (Tabelle 1) sowie die daraus resultierende Korrelation neu berechnet (Abbildung 2).

Aus der Korrelationsgeradengleichung (Abbildung 2) ergibt sich für den BAT-Wert von 15 mg Methoxyessigsäure/g Kreatinin eine Luftkonzentration von 2,4 ml 2-Methoxyethanol/m^3^.

**Tab. 1:** Arbeitsmedizinische Studien mit Erfassung der äußeren Exposition gegen 2-Methoxyethanol und der Ausscheidung an Methoxyessigsäure im Urin (geometrische Mittelwerte mit geometrischer Standardabweichung, GSD)

2-Methoxyethanol in der Luft [ml/m^3^]	Methoxyessigsäure im Urin [mg/g Kreatinin]	Bemerkung	Literatur
2,14; GSD 2,01	5,44; GSD 3,59 (n = 49)	Kontrollgruppe: unterhalb der Nachweisgrenze, 82 % der Beschäftigten ohne Handschuhe; 18 % mit Baumwollhandschuhen	Chang et al. 2004
8,13; GSD 1,62	72,63; GSD 2,04 (n = 25)	60 % der Beschäftigten mit Baumwollhandschuhen, 12 % mit Butylgummihandschuhen	
nicht detektierbar bis 0,28 (Kontrollwert nicht in Korrelation beinhaltet)	1,26; GSD 1,62 (n = 32)	Kontrollgruppe	Shih et al. 2000
3,98; GSD 2,88	19,95; GSD 2,19 (n = 30) (Fabrik 1)	k. A. zu Schutzmaßnahmen	
4,27; GSD 2,19	20,89; GSD 2,19 (n = 15) (Fabrik 2)		
0,08; GSD 5,09	0,53; GSD 3,40 (n = 32)	Gruppe ohne Exposition/Kontrollgruppe	Shih et al. 2003
0,34; GSD 2,69	6,77; GSD 4,19 (n = 29), Zeitpunkt 2	Abzüge installiert; Anweisung an Beschäftigte, Atemschutz u. Gummihandschuhe zu tragen	
2,34; GSD 1,76	19,7; GSD 2,09 (n = 29), Zeitpunkt 1		
9,62; GSD 4,75	50,7; GSD 1,67 (n = 29)	vor Expositionsminderungsmaßnahmen	

MAA: Methoxyessigsäure; ME: Methoxyethanol

#### 3.1.2 Ratte und Maus

Für männliche bzw. weibliche Ratten wird eine durchschnittliche Eliminationshalbwertszeit im Blut von 12,6 und 14,1 Stunden berichtet, errechnet aus den Urinmessungen von Methoxyessigsäure nach einmaliger intraperitonealer Gabe von 100 mg 2-Methoxyethanol/kg KG (Aasmoe und Aarbakke 1997).

In einer Toxikokinetikstudie wurden Blut- und Urinwerte von trächtigen Sprague-Dawley-Ratten nach inhalativer Ganzkörperexposition gegen 10 (NOAEC für Entwicklungstoxizität) und 50 ml 2-Methoxyethanol/m^3^ für sechs Stunden pro Tag vom 11. bis zum 15. Gestationstag bestimmt. Die höchsten Blutkonzentrationen betrugen 7,1 bzw. 62,7 mg Methoxyessigsäure/l (0,08 bzw. 0,7 mM). Es wurde ein Physiologie-basiertes pharmakokinetisches Modell (PBPK-Modell) entwickelt, aus dem hervorgeht, dass eine Luftkonzentration von 10 ml 2-Methoxyethanol/m^3^ bei der trächtigen Ratte einer Luftkonzentration beim Menschen von 12 ml 2-Methoxyethanol/m^3^ entspricht. Das Modell beinhaltet die Extrapolation von sechs auf acht Stunden (für den Arbeitsplatz) und berücksichtigt die Unterschiede der Halbwertszeiten bei Ratte (12,6 und 14,1 Stunden) und Mensch (77 Stunden) (Gargas et al. 2000).

## 4 Erfahrungen beim Menschen

### Wiederholte Exposition

Hierzu liegen seit dem Nachtrag aus dem Jahr 2009 keine neuen Daten vor.

### Allergene Wirkung

Es liegen weiterhin keine Daten vor.

### Reproduktionstoxizität

#### Fertilität

Im Nachtrag aus dem Jahr 2009 (Hartwig 2009) und im BAT-Addendum aus dem Jahr 2009 (Käfferlein 2009) werden in mehreren Arbeitsplatzstudien mit Exposition gegen 2-Methoxyethanol sich widersprechende Ergebnisse zu Spermienmorphologie und -anzahl berichtet. Quantitative Angaben zu den entsprechenden Expositionssituationen sind entweder nicht vorhanden oder lückenhaft und die Beschäftigten waren zusätzlich gegen mehrere Lösungsmittel gleichzeitig oder gegen weitere Verbindungen aus der Substanzklasse der Glykolether exponiert.

Neue Studien sind zu diesem Endpunkt seit dem Nachtrag aus dem Jahr 2009 nicht veröffentlicht worden.

#### Entwicklungstoxizität

Im Nachtrag aus dem Jahr 2009 (Hartwig 2009) und im BAT-Addendum aus dem Jahr 2024 (Michaelsen et al. 2024) werden mehrere Studien zur Untersuchung des Einflusses einer beruflichen Exposition gegen Glykolether auf das Auftreten von kongenitalen Fehlbildungen berichtet. Im BAT-Addendum wird beschrieben, dass konsistente und qualitativ nachvollziehbare Berichte über eine positive Assoziation zwischen Aborten und einer Exposition gegen Glykolethern in der Halbleiterindustrie vorliegen (Käfferlein 2009). Jedoch sind die Studien aufgrund von Mischexposition, fehlender Expositionserfassung, mangelhafter Methodik oder Dokumentation nicht zur Bewertung entwicklungstoxischer Effekte von 2-Methoxyethanol geeignet.

## 5 Tierexperimentelle Befunde und In-vitro-Untersuchungen

### 5.1 Akute Toxizität

Es liegen keine neuen Daten vor.

### 5.2 Subakute, subchronische und chronische Toxizität

Zahlreiche Studien am Tier belegen als Zielorgane der 2-Methoxyethanol-induzierten Toxizität, unabhängig von der Applikationsart, die blutbildenden und lymphatischen Organe, das Blut, die Hoden und in geringerem Maße Nieren, Leber und Nervensystem (Hartwig 2009). Auch die Ovarien bei Ratten haben sich in seit dem letzten Nachtrag veröffentlichten Studien als mögliches Ziel der Toxizität herausgestellt (siehe Abschnitt 5.5.1).

#### 5.2.1 Inhalative Aufnahme

In einer Studie mit 13-wöchiger Inhalation (6 h/Tag, 5 Tage/Woche) sind die männlichen Reproduktionsorgane bei Sprague-Dawley-Ratten und bei Neuseeländer-Kaninchen die empfindlichsten Organe (siehe auch Abschnitt 5.5.1; Hartwig 2009).

#### 5.2.2 Orale Aufnahme

Eine 13-wöchige Trinkwasserstudie des NTP ergab einen LOAEL für Effekte auf Hoden, Thymus und Blut von 70 mg/kg KG und Tag (niedrigste Dosis) bei F344-Ratten und einen NOAEL für Effekte auf Hoden und Milz von 300 mg/kg KG bei B6C3F1-Mäusen. Männliche Tiere waren jeweils empfindlicher als weibliche Tiere (Hartwig 2009; NTP 1993).

Seit dem Nachtrag von 2009 liegt nur eine neue Studie mit wiederholter oraler Gabe vor.

An Mäusen wurde die protektive Wirkung von Xanthon als Antioxidans bei der 2-Methoxyethanol-induzierten Herzschädigung untersucht. Dazu wurden acht männlichen Mäusen (k. A. zum Stamm) pro Gruppe fünf Wochen lang per Gavage 0 oder 200 mg 2-Methoxyethanol/kg KG und Tag verabreicht. Die Gabe von 2-Methoxyethanol führte zu nekrotischen Veränderungen am Herzen, die mit Lipidperoxidation einhergingen (Ernawati et al. 2019). Es wurde nur eine Dosis getestet, so dass die Angabe eines NOAEL nicht möglich ist.

#### 5.2.3 Dermale Aufnahme

Es liegen keine neuen Daten vor.

### 5.3 Wirkung auf Haut und Schleimhäute

Es liegen keine neuen Daten vor.

### 5.4 Allergene Wirkung

Es liegen keine neuen Daten vor. Aufgrund der Molekülstruktur besteht kein Verdacht auf eine sensibilisierende Wirkung.

### 5.5 Reproduktionstoxizität

#### 5.5.1 Fertilität

##### 5.5.1.1 Männliche Fertilität

Für 2-Methoxyethanol ist die toxische Wirkung auf die Hoden gut dokumentiert. Der Stoff führt bei Ratten, Mäusen, Kaninchen und Affen nach inhalativer, oraler und dermaler Gabe zu Schäden am Keimepithel der Hoden (ECETOC 2005 a, b; Sakurai et al. 2015). Alle Spermatogenesestadien sind von der Wirkung betroffen. Bei Ratten ist gezeigt worden, dass die Pachytänspermatozyten (besonders die Stufen VII und VIII) am sensitivsten und vorwiegend von Schäden betroffen sind. Die Leydig-Zellen, die für die Testosteronproduktion verantwortlich sind, sind nicht beeinträchtigt. Der Effekt wird über den Metaboliten Methoxyessigsäure vermittelt (ECETOC 2005 a, b).

Im Nachtrag aus dem Jahr 2009 (Hartwig 2009) wird berichtet, dass bei Sprague-Dawley-Ratten nach 13-wöchiger Inhalation ab 300 ml 2-Methoxyethanol/m^3^ bilaterale diffuse, moderate bis schwere Degenerationen des Keimepithels in den Samenkanälchen (NOAEC 100 ml 2-Methoxyethanol/m^3^) und bei Neuseeländer-Kaninchen ab 30 ml 2-Methoxyethanol/m^3^ degenerative Veränderungen des Hodens (niedrigste Dosis, beginnende Effekte bei einem von fünf Tieren) auftreten. Bei Gabe mit dem Trinkwasser kommt es bei männlichen Ratten ab 220 mg 2-Methoxyethanol/kg KG und Tag (NOAEL 87 mg 2-Methoxyethanol/kg KG und Tag) nach 10-tägiger Verabreichung zu erniedrigtem Hodengewicht und testikulären Schäden und bei männlichen Kaninchen ab 25 mg 2-Methoxyethanol/kg KG und Tag (NOAEL 12,5 mg 2-Methoxyethanol/kg KG und Tag) zu einer gestörten Spermatogenese nach 12-wöchiger Exposition.

Auch die seit dem Jahr 2009 veröffentlichten Studien bestätigen die Toxizität von 2-Methoxyethanol auf Hoden und Spermien von Ratten und Affen (siehe Tabelle 2; Adeyemo-Salami und Farombi 2018; Sakurai et al. 2015; Starek-Świechowicz et al. 2015). Bei Ratten traten dabei die Effekte nach 2-wöchiger Gavage-Gabe und 4-wöchiger subkutaner Gabe jeweils ab etwa 100 mg/kg KG und Tag (Adeyemo-Salami und Farombi 2018; Sakurai et al. 2015; Starek-Świechowicz et al. 2015) und bei Affen nach Gabe per Gavage bei 300 mg/kg KG und Tag (einzige Dosis) (Sakurai et al. 2015) auf.

##### 5.5.1.2 Weibliche Fertilität

Eine Fertilitätsstudie an SD (Crl:CD(SD))-Ratten mit Gavage-Gabe zwei Wochen vor der Verpaarung, zwei Wochen während der Paarungszeit und bis zum 6. Gestationstag an weibliche Tiere, die mit unbehandelten männlichen Tieren verpaart wurden, führte ab der niedrigsten Dosis von 30 mg/kg KG und Tag zu einer geringeren mittleren Anzahl von Implantationen und lebenden Embryos sowie einem erhöhten gesamten mittleren Postimplantationsverlust (Dodo et al. 2009).

Studien, die seit dem letzten Nachtrag publiziert worden sind, zeigen die toxische Wirkung von 2-Methoxyethanol auf die Ovarien von Ratten (Tabelle 2; Dodo et al. 2009; Taketa et al. 2011 a, b, 2013). So kam es nach 2- oder 4-wöchiger Gavage-Gabe bei SD (Crl:CD(SD))-Ratten ab 100 mg/kg KG und Tag zu einer Hypertrophie der Corpora lutea, verlängerten Östruszyklen (kontinuierlicher Diöstrus) und einer Inhibierung der Ovulation. Der NOAEL lag bei 30 mg/kg KG und Tag (Dodo et al. 2009; Taketa et al. 2011 a).

**Tab. 2:** Tierstudien (publiziert seit dem letzten Nachtrag aus dem Jahr 2009) zur Wirkung auf die Reproduktionsorgane: NOAEL und LOAEL nach oraler oder subkutaner Verabreichung von 2-Methoxyethanol

Spezies, Stamm, Tierzahl, Geschlecht, Exposition, Dauer Untersuchte Organe/Gewebe	NOAEL	LOAEL	Literatur
**Männliche Reproduktionsorgane**			
**Ratte**			
Wistar, 10 ♂, oral (Gavage), 2 Wo Hoden, Nebenhoden (Organgew. ↓, Histologie), Spermien (reduzierte Anzahl)	–	100 mg/kg KG u. d	Adeyemo-Salami und Farombi 2018
Wistar, 5 ♂, subkutan, 4 Wo Hoden (rel. Organgew. ↓, Atrophie), Nebenhoden (rel. Organgew. ↓)	–	95 mg/kg KG u. d	Starek-Świechowicz et al. 2015
**Affe**			
Cynomolgus, 3 ♂, oral (Gavage), 4 d Hoden (Abnahme der Pachytänspermatozyten Stufe I–VI u. der Spermatiden Stufe I–IV, Vakuolisierungen in den Samenkanälchen)	–	300 mg/kg KG u. d (einzige Dosis)	Sakurai et al. 2015
**Weibliche Reproduktionsorgane**			
**Ratte**			
SD (Crl:CD(SD)), 10 ♀, oral (Gavage), Fertilitätsstudie: 2 Wo vor Verpaarung u. 2 Wo Paarungszeit u. bis GD 6 (♂ unbehandelt) Mittlere Anzahl an Implantationen ↓, mittlere Anzahl lebender Embryos ↓, gesamter mittlerer Postimplantationsverlust ↑	–	30 mg/kg KG u. d	Dodo et al. 2009
SD (Crl:CD(SD)), 10 ♀, oral (Gavage), 2 od. 4 Wo Corpora lutea (Hypertrophie), verlängerter Östruszyklus (kontinuierlicher Diöstrus), Inhibierung der Ovulation	30 mg/kg KG u. d	100 mg/kg KG u. d	Dodo et al. 2009
SD (Crl:CD), 5‑8 ♀, oral (Gavage), 2 Wo Corpora lutea (Histologie)	–	300 mg/kg KG u. d (einzige Dosis)	Taketa et al. 2011 b
Crl:CD(SD), 10 ♀, oral (Gavage), 2 od. 4 Wo Corpora lutea (Hypertrophie), verlängerter Östruszyklus (kontinuierlicher Diöstrus)	30 mg/kg KG u. d	100 mg/kg KG u. d	Taketa et al. 2011 a
SD (Crl:CD), 4‑5 ♀, oral (Gavage), 7 d Corpora lutea (Hypertrophie)	–	300 mg/kg KG u. d (einzige Dosis)	Taketa et al. 2013

d: Tag; Wo: Woche

#### 5.5.2 Entwicklungstoxizität

Zu 2-Methoxyethanol liegt eine Vielzahl von Entwicklungstoxizitätsstudien vor. Eine Übersicht ist bei ECETOC (2005 a) dargestellt. Die bewertungsrelevanten Studien (Inhalation bis 25 ml/m^3^, orale Aufnahme bis 50 mg/kg KG und Tag) sind in Tabelle 3 aufgeführt.

Methoxyethanol führt bei Ratten, Mäusen, Kaninchen und Affen nach inhalativer, oraler und dermaler Gabe zu entwicklungstoxischen, einschließlich teratogenen Effekten. Nach **Inhalation** liegen die LOAEC für Entwicklungstoxizität bei 25 ml 2-Methoxyethanol/m^3^ für Ratten (Driscoll et al. 1998) bzw. 50 ml 2-Methoxyethanol/m^3^ für Mäuse (Hanley et al. 1984) und die entsprechenden NOAEC bei 10 ml 2-Methoxyethanol/m^3^ für beide Spezies (Hanley et al. 1984). Für das Kaninchen leiten die Autoren eine NOAEC von 10 ml 2-Methoxyethanol/m^3^ ab. Bei dieser Konzentration treten jedoch statistisch signifikant erhöhte Prozentsätze an resorbierten Implantationen und an Würfen mit Resorptionen sowie an verzögerten Ossifikationen des Brustbeins auf. Die Prozentsätze der resorbierten Implantationen (Kontrolle: 4 % (7/180); 3 ml/m^3^: 8 % (14/186); 10 ml/m^3^: 11 % (23/210)* (*signifikant unterschiedlich zum Kontrollwert); 50 ml/m^3^: 24 % (46/191)*; historische Kontrollen des Labors: 9 ± 4 %; Bereich: 4–18 %) und Würfe mit Resorptionen (Kontrolle: 22 % (5/23); 3 ml/m^3^: 42 % (10/24); 10 ml/m^3^: 58 % (14/24)*; 50 ml/m^3^: 67 % (16/24)*; historische Kontrollen des Labors: 39 ± 13 %; Bereich: 15–67 %) liegen bei 10 ml/m^3^ im Bereich der historischen Kontrolle des Labors. Die verzögerten Ossifikationen (Fetenbasis (Wurfbasis), Kontrolle: 82/173 (23/23); 3 ml/m^3^: 93/172 (23/23); 10 ml/m^3^: 123/187 (23/24)*; 50 ml/m^3^: 127/143 (22/22)*) sehen die Autoren im Bereich der „normalen“ Variabilität dieser Spezies ohne dies weiter auszuführen (Hanley et al. 1984). Nach Ansicht der Kommission handelt es sich um einen Grenzfall. Da bei 10 ml/m^3^ Effekte nicht auszuschließen sind, keine Maternaltoxizität auftritt und der Stoff zu einer bekannten Gruppe teratogener Stoffe gehört, wird die NOAEC für Entwicklungstoxizität für das Kaninchen in dieser Studie konservativ bei 3 ml/m^3^ festgesetzt (LOAEC 10 ml/m^3^).

Die LOAEL für Entwicklungstoxizität nach **oraler** Gabe liegen für Ratten und Mäuse bei 25 bzw. 31 mg 2-Methoxyethanol/kg KG und Tag (Nagano et al. 1981; Nelson et al. 1989; Toraason et al. 1985) bzw. für Affen bei 12 mg 2-Methoxyethanol/kg KG und Tag (Scott et al. 1989). Für Ratten liegt der orale NOAEL für Teratogenität bei 16 mg 2-Methoxyethanol/kg KG und Tag (Nelson et al. 1989), während ein NOAEL für Entwicklungstoxizität für die Spezies Maus und Affe nicht ableitbar ist (Tabelle 4.1.3 in ECETOC 2005 b; Nagano et al. 1981; Scott et al. 1989). Für Kaninchen liegen keine oralen Untersuchungen zur Entwicklungstoxizität vor. In Untersuchungen zur Reproduktionstoxizität mittels kontinuierlicher Verpaarung (Reproductive Assessment by Continous Breeding) an Ratten hat sich nach Trinkwassergabe ein NOAEL von etwa 15 mg 2-Methoxyethanol/kg KG und Tag ergeben, wenn die 2. Generation mit dem 5. Wurf der 1. Generation erzeugt wurde. Wenn jedoch der 2. Wurf zur Erzeugung der 2. Generation verwendet wurde, konnte kein NOAEL für eine erniedrigte Anzahl lebender Nachkommen in der F1-Generation abgeleitet werden (Environmental Health Research and Testing Inc. 1990 a, b; Gulati et al. 1991), sodass sich hier insgesamt kein NOAEL für eine erniedrigte Anzahl lebender Nachkommen ableiten lässt. Jedoch ist zu dieser Studie anzumerken, dass aufgrund des Effektes von 2-Methoxyethanol auf die In-utero-Entwicklung und die paternalen Hoden/Spermien die Fetotoxizität mit der Fertilität interferieren kann (ECETOC 2005 b).

Bei Wistar-Ratten ist es nach **dermaler** okklusiver Applikation vom 6. bis zum 15. Gestationstag ab der niedrigsten Dosis von 50 mg 2-Methoxyethanol/kg KG und Tag vermehrt zu Fehlbildungen sowie Embryo- und Fetotoxizität gekommen. Ein NOAEL konnte nicht abgeleitet werden (Hellwig 1993 in ECETOC 2005 b). Dieser unveröffentlichte Studienbericht lag der Kommission nicht vor.

Bei Wistar-Ratten haben sich nach oraler Gabe am 12. Gestationstag 2-Methoxyethanol und Methoxyessigsäure auf äquimolarer Basis als ähnlich potent hinsichtlich der Auslösung von Defekten an Herz, Schwanz und Gliedmaßen sowie Hydronephrose erwiesen. So liegt der Anteil an Feten mit Defekten der Gliedmaßen bei 9,3 % bei 2,07 mmol (158 mg/kg KG) 2-Methoxyethanol und bei 60,6 % bei 4,14 mmol (315 mg/kg KG) 2-Methoxyethanol. Nach Gabe von 2,07 mmol (190 mg/kg KG) und 4,14 mmol (380 mg/kg KG) Methoxyessigsäure beträgt der Anteil 14,4 % bzw. 69,1 % (Ritter et al. 1985).

Für trächtige Mäuse wurde berichtet, dass maternale Plasmakonzentrationen von Methoxyessigsäure von mehr als 1 mM zu Entwicklungstoxizität führen (Welsch et al. 1995).

**Tab. 3:** Bewertungsrelevante Entwicklungstoxizitätsstudien nach Verabreichung von 2-Methoxyethanol

Spezies, Stamm, Anzahl pro Gruppe	Exposition	Befunde	Literatur
Inhalativ			
**Ratte**, F344, 30‑31 ♀	**GD 6–15**, 0, 3, 10, 50 ml/m^3^, 6 h/d, Untersuchung GD 21, **ähnlich OECD TG 414**	**keine NOAEC für Maternaltoxizität**; **ab 3 ml/m^3^**: Muttertiere: Hämoglobin ↓, Hämatokrit ↓; **10 ml/m^3^**:** NOAEC für Entwicklungstoxizität**; **50 ml/m^3^**: Muttertiere: Erythrozytenzahl ↓, abs. Lebergew. ↑, Feten: skelettale Variationen ↑	Hanley et al. 1984
**Ratte**, Crl:CD BR, 25‑26 ♀	**GD 7–16**, 0, 25 ml/m^3^, Einsatz als Positivkontrolle, 6 h/d, über die Nase, Untersuchung GD 22	**25 ml/m^3^**: Muttertiere: rel. Lebergew. ↑, Futterverbrauch ↓, Feten: verzögerte Ossifikationen (Schädelknochen, v. a. Interparietale, Parietale, Supraoccipitale), skelettale Variationen ↑ (rudimentäre Lumbalrippen)	Driscoll et al. 1998
**Maus**, CF-1, 30‑32 ♀	**GD 6–15**, 0, 10, 50 ml/m^3^, 6 h/d, Untersuchung GD 18, **ähnlich OECD TG 414** (eine Konzentration weniger)	**10 ml/m^3^**:** NOAEC für Entwicklungs- u. Maternaltoxizität**; **50 ml/m^3^**: Muttertiere: KG-Zunahme ↓, Feten: skelettale Variationen ↑, einseitige testikuläre Hypoplasie	Hanley et al. 1984
**Kaninchen**, Weiße Neuseeländer, 29‑30 ♀	**GD 6–18**, 0, 3, 10, 50 ml/m^3^, 6 h/d, Untersuchung GD 29, **ähnlich OECD TG 414**	**3 ml/m^3^: NOAEC für Entwicklungstoxizität (siehe Text)**; **10 ml/m^3^: NOAEC für Maternaltoxizität**; **ab 10 ml/m^3^**: Feten: resorbierte Implantationen ↑ (Kontrolle: 4 % (7/180); 3 ml/m^3^: 8 % (14/186); 10 ml/m^3^: 11 % (23/210)*; 50 ml/m^3^: 24 % (46/191)*; historische Kontrollen des Labors: 9 ± 4 %; Bereich: 4–18 %), Anteil der Würfe mit Resorptionen ↑ (Kontrolle: 22 % (5/23); 3 ml/m^3^: 42 % (10/24); 10 ml/m^3^: 58 % (14/24)*; 50 ml/m^3^: 67 % (16/24)*; historische Kontrollen des Labors: 39 ± 13 %; Bereich: 15–67 %), reduzierte Ossifikationen der Sternebrae: Fetenbasis (Wurfbasis), Kontrolle: 82/173 (23/23); 3 ml/m^3^: 93/172 (23/23); 10 ml/m^3^: 123/187 (23/24)*; 50 ml/m^3^: 127/143 (22/22)*); **50 ml/m^3^**: Muttertiere: KG-Zunahme ↓ (bei statistisch signifikant höherem KG am 6. GD), abs. Lebergew. ↑, Feten: KG ↓, viszerale Fehlbildungen ↑ (besonders betroffen: Herz, Milz, Niere), skelettale Fehlbildungen ↑, skelettale u. viszerale Variationen ↑	Hanley et al. 1984
Oral			
**Ratte**, Sprague Dawley, 9‑12 ♀	**GD 7–18**, 0; 0,006; 0,012; 0,025; 0,05; 0,1; 0,25; 0,5 % in Flüssignahrung (0, 16, 31, 73, 140, 198, 290, 620 mg/kg KG u. d), Untersuchung GD 20, **ähnlich OECD TG 414** (geringere Tierzahl, mehr Dosierungen, einzelne Variationen bzw. Fehlbildungen nicht dargestellt, nur Gesamtzahl)	**16 mg/kg KG**:** NOAEL für Teratogenität**; **ab 16 mg/kg KG**: Feten: KG ↓ (nicht auf Wurf bezogen); **31 mg/kg KG**:** NOAEL für Maternaltoxizität**; **ab 31 mg/kg KG**: Feten: viszerale u. skelettale Fehlbildungen ↑ (doppelte u./od. fehlplatzierte Aorten u./od. ventrikuläre Septumdefekte; fusionierte Rippen, fehlende Wirbel); **ab 140 mg/kg KG**: Muttertiere: KG-Zunahme ↓, Feten: 100 % Mortalität	Nelson et al. 1989
**Ratte**, Sprague Dawley, Dosisgruppen: 8 ♀, Kontrollgruppe: 11 ♀	**GD 7–13**, 0, 25, 50, 100 mg/kg KG u. d, Gavage, Untersuchung GD 20, Fokus auf Untersuchung des Herzens	**kein NOAEL für Entwicklungstoxizität**; **25 mg/kg KG**: Feten: EKG: verlängerte QRS-Welle (Hinweis auf intraventrikuläre Leitungsstörung); **50 mg/kg KG**:** NOAEL für Maternaltoxizität**; Feten: kardiovaskuläre Fehlbildungen ↑ (v. a. ventrikuläre Septumdefekte u. rechter Ductus arteriosus); **100 mg/kg KG**: Muttertiere: 100 % Resorptionen	Toraason et al. 1985
**Ratte**, VAF Crl: CD BR Auszucht Sprague Dawley, Dosisgruppen: 20 ♀ u. 20 ♂, Kontrollgruppe: 40 ♀ u. 40 ♂	**Reproductive Assessment by Continous Breeding**, 2 Generationen, ca. 20 Wo Exposition; Entwicklung eines Protokolls für Ratten; zur Generierung der 2. Generation: 2. Wurf der 1. Generation verwendet: 0; 0,01; 0,03; 0,1 % im Trinkwasser (0; F0: ♀: 12,7; 36,3; 122,1 mg/kg KG u. d, ♂: 8,8; 23,6; 75,8 mg/kg KG u. d; F1: ♀: 15,0; 40,8 mg/kg KG u. d, ♂: 9,1; 27,2 mg/kg KG u. d); zur Generierung der 2. Generation: 5. Wurf der 1. Generation verwendet: 0; 0,006; 0,012; 0,024 % im Trinkwasser (0; F0: ♀: 7,3; 15,3; 32,7 mg/kg KG u. d, ♂: 5,0; 9,6; 20,9 mg/kg KG u. d; F1: ♀: 7,1; 14,2; 24,6 mg/kg KG u. d, ♂: 3,9; 8,1; 15,6 mg/kg KG u. d)	2. Wurf: **15,0 mg/kg KG**: F1: Anzahl lebender Nachkommen ↓; **36,3 mg/kg KG**: F0: Anzahl lebender Nachkommen/Wurf ↓ (♂, ♀, kombiniert); **122,1 mg/kg KG**: F0: Fertilitätsindex ↓ (5 %, Kontrolle: 100 %); 5. Wurf: **15,3/14,2 mg/kg KG**:** NOAEL (F0/F1 ♀)**; **32,7/24,6 mg kg/KG**: F0, F1: Anzahl lebender Nachkommen/Wurf ↓ Fetotoxizität kann mit der Fertilität interferieren (ECETOC 2005 b): siehe Abschnitt 5.5.1 (ausgeprägte Hodentoxizität)	Environmental Health Research and Testing Inc. 1990 a, b; Gulati et al. 1991
**Maus**, ICR, 21‑24 ♀	**GD 7–14**, 0, 31, 63, 125, 250, 500, 1000 mg/kg KG u. d, Gavage, Vehikel: entionisiertes Wasser, Untersuchung GD 18, Fokus auf externe u. skelettale Untersuchung	**kein NOAEL für Entwicklungstoxizität**; **ab 31 mg/kg KG**: Feten: skelettale Variationen ↑ (gegabelte od. gespaltene Halswirbelsäule); **125 mg/kg KG: NOAEL für Maternaltoxizität**; **ab 125 mg/kg KG**: Feten: KG ↓; **ab 250 mg/kg KG**: Muttertiere: KG-Zunahme ↓, Feten: Mortalität ↑, externe Fehlbildungen (Exenzephalie, Oligodaktylie, Nabelhernien), skelettale Fehlbildungen (fusionierte Rippen, fusionierte od. fehlende Wirbel, Spina bifida occulta); **500 mg/kg KG**: Feten: nur ein lebender Fetus (mit Exenzephalie, fehlgebildete Finger); **1000 mg/kg KG**: Muttertiere: Leukopenie, Feten: 100 % Mortalität	Nagano et al. 1981
**Affe**, Macaca fascicularis, Dosisgruppen: 8‑14 ♀, Kontrollgruppe: 6 ♀, Ethanolgruppe: 3 ♀	**GD 20–45**, 0, 12, 24, 36 mg/kg KG u. d, Gavage, Vehikel: Wasser, Kontrollgruppen: Gruppe 1: ohne Gavagebehandlung, Gruppe 2: Gavage mit gleichen Volumen an Ethanol, Untersuchung GD 100	**kein NOAEL für Entwicklungs- u. Maternaltoxizität**; **ab 12 mg/kg KG**: Muttertiere: leichte Anorexie; **12 mg/kg KG**: Feten: 4/14 intrauteriner Tod; **24 mg/kg KG**: Muttertiere: Anorexie u. daher Gavagegabe von Brei u./od. Elektrolyten, Feten: 3/11 intrauteriner Tod (1 davon: Spontanabort); **36 mg/kg KG**: Muttertiere: schwere Anorexie u. daher Gavagegabe von Brei u./od. Elektrolyten, Feten: 8/8 intrauteriner Tod, ein Fetus mit externen Fehlbildungen (Fehlen jeweils eines Fingers an beiden Vordergliedmaßen); Halbwertszeit von Methoxyessigsäure im Serum der Muttertiere ca. 20 h	Scott et al. 1989
Dermal			
**Ratte**, Wistar, 45‑50 ♀	**GD 6–15**, 0, 50, 100, 290, 480, 770, 970 mg/kg KG u. d, okklusiv, 6 h/d, Untersuchung GD 20, **vermutlich ähnlich OECD TG 414**	**kein NOAEL für Entwicklungstoxizität**; **50 mg/kg KG**: Feten: Fehlbildungen ↑, Feto- u. Embryotoxizität; **100 mg/kg KG**: Muttertiere: KG-Zunahme ↓, Postimplantationsverluste (26,5 %), Feten: Fehlbildungen ↑; **290 mg/kg KG**: Muttertiere: Postimplantationsverluste (99,4 %), Feten: 5 fehlgebildete Feten; **ab 480 mg/kg KG**: Muttertiere: KG ↓, keine Würfe, Resorptionen 100 %	Hellwig 1993 zitiert in ECETOC 2005 b, Studienbericht nicht vorliegend

*statistisch signifikant

d: Tag; GD: Gestationstag; PND: Postnataltag; QRS: Kammerkomplex, Bestandteil des Elektrokardiogramms; TG: Test Guideline (Prüfrichtlinie)

**Fazit zur Entwicklungstoxizität**: 2-Methoxyethanol besitzt unter den Glykolethern die höchste entwicklungstoxische Potenz. Die Wirkung wird über den Metaboliten Methoxyessigsäure vermittelt. Teratogene Effekte betreffen Skelett sowie innere Organe, wobei Fehlbildungen von Rippen und Wirbelkörpern sowie kardiovaskuläre Fehlbildungen im Vordergrund stehen. Bei höheren Dosierungen kommt es zum intrauterinen Tod der Embryos bzw. Feten. Die bewertungsrelevanten NOAEC/NOAEL und LOAEC/LOAEL für Entwicklungstoxizität bei Ratte, Maus, Kaninchen und Affe sind in Tabelle 4 dargestellt.

**Tab. 4:** Bewertungsrelevante NOAEC/NOAEL und LOAEC/LOAEL für Entwicklungstoxizität nach Verabreichung von 2-Methoxyethanol

Endpunkt, Exposition	NOAEC/NOAEL	LOAEC/LOAEL	Literatur
**Ratte**			
pränatal, inhalativ	10 ml/m^3^	50 ml/m^3^	Hanley et al. 1984
pränatal, inhalativ	–	25 ml/m^3^	Driscoll et al. 1998
pränatal, Flüssignahrung	16 mg/kg KG u. d	31 mg/kg KG u. d	Nelson et al. 1989
pränatal, Gavage	–	25 mg/kg KG u. d	Toraason et al. 1985
pränatal, dermal-okklusiv	–	50 mg/kg KG u. d	Hellwig 1993 in ECETOC 2005 b
**Maus**			
pränatal, inhalativ	10 ml/m^3^	50 ml/m^3^	Hanley et al. 1984
pränatal, Gavage	–	31 mg/kg KG u. d	Nagano et al. 1981
**Kaninchen**			
pränatal, inhalativ	3 ml/m^3^	10 ml/m^3^	Hanley et al. 1984
**Makake**			
pränatal, Gavage	–	12 mg/kg KG u. d	Scott et al. 1989

d: Tag

### 5.6 Genotoxizität

Im Nachtrag aus dem Jahr 2009 wurde die Datenlage zur Genotoxizität von 2-Methoxyethanol ausführlich dargestellt. Neben negativen Ergebnissen in Genotoxizitätstests gibt es auch Studien, die klastogene Effekte in vitro bei hohen Konzentrationen von 65 mM (5 mg/ml) und positive Effekte in vivo in Indikatortests zeigen; chromosomale Aberrationen und dominante Letalmutationen sind in vivo jedoch nicht induziert worden (Hartwig 2009).

#### 5.6.1 In vitro

2-Methoxyethanol führte in einem Comet-Assay an 3D-kultivierten HepG2-Zellen ohne Zusatz eines metabolischen Aktivierungssystems bis zu einer Konzentration von 10 mM (0,8 mg/ml) nicht zu DNA-Schäden. Mit Zusatz eines metabolischen Aktivierungssystems hatte der Stoff ab 2,5 mM (0,2 mg/ml) eine konzentrationsabhängige Erhöhung der %Tail-DNA-Intensität zur Folge. Zytotoxizität wurde bis 10 mM nicht beobachtet (Lim et al. 2022). Es ist nicht nachvollziehbar, wie viele Replikate untersucht wurden.

#### 5.6.2 In vivo

Hierzu liegen seit dem Nachtrag aus dem Jahr 2009 keine neuen Daten vor.

#### 5.6.3 Fazit zur Genotoxizität

2-Methoxyethanol induziert in vitro klastogene Effekte bei hohen Konzentrationen. Diese sind jedoch in vivo im Dominant-Letal-Test, im Mikronukleustest und im Chromosomenaberrationstest nicht bestätigt worden (Hartwig 2009).

### 5.7 Kanzerogenität

Es liegen weiterhin keine Daten vor. Ein Strukturverdacht besteht nicht.

## 6 Bewertung

Kritische Effekte sind die Veränderung hämatologischer Parameter beim Menschen (Hartwig 2009) und die Effekte auf männliche Reproduktionsorgane sowie die entwicklungstoxische Wirkung im Tierexperiment.

**MAK-Wert. **Im Nachtrag aus dem Jahr 2009 wurde eine Korrelation der Luftkonzentration von 2-Methoxyethanol und der Urinkonzentration des Metaboliten Methoxyessigsäure aus arbeitsmedizinischen Studien dargestellt. Diese wurde als Basis für die Ableitung des MAK-Wertes von 2-Methoxyethanol verwendet (Hartwig 2009). Die in diesem Nachtrag korrigierte Korrelationsgerade (Abbildung 2) ergibt ausgehend vom BAT-Wert von 15 mg Methoxyessigsäure/g Kreatinin eine Luftkonzentration von 2,4 ml 2-Methoxyethanol/m^3^. Die Korrelation wird nicht zur Ableitung eines MAK-Wertes verwendet, da nicht bekannt ist, ob und wie sich die Expositionsminimierungsmaßnahmen auf die innere Belastung der Exponierten ausgewirkt haben.

Im BAT-Addendum von 2024 (Michaelsen et al. 2024) wurde bei der Vergabe eines Hinweises auf Voraussetzung für Schwangerschaftsgruppe C die Umrechnung einer Luftkonzentration von 2-Methoxyethanol in eine Urinkonzentration der Methoxyessigsäure auf der Basis einer Probandenstudie (Groeseneken et al. 1989) und toxikokinetischen Überlegungen vorgenommen. Die Probanden sind inhalativ gegen 5 ml 2-Methoxyethanol/m^3^ exponiert gewesen (über Atemmaske unter Ruhebedingungen; Groeseneken et al. 1989). Die Exposition von 4 × 50 min (200 min) entspricht einer mittleren Gesamtaufnahme von 19,4 mg 2-Methoxyethanol, wovon nach 24 h 15,3 % (ca. 3 mg) als Methoxyessigsäure ausgeschieden werden (siehe Abschnitt 3.1). Umgerechnet auf eine 8-stündige Exposition entspricht dies 7,2 mg Methoxyessigsäure (3 mg Methoxyessigsäure × 480 min/200 min). Unter Berücksichtigung, dass dieselbe Menge zusätzlich jeweils über die Haut und über das erhöhte Atemvolumen aufgenommen wird (insgesamt 21,6 mg), ergibt sich eine Ausscheidung nach 8-stündiger Exposition von 7,2 mg Methoxyessigsäure (21,6 mg Methoxyessigsäure × 8/24). Umgerechnet auf Kreatinin ergibt sich eine Ausscheidung von 16,7 mg Methoxyessigsäure/g Kreatinin, da die Kreatinin-Ausscheidung bei 1,3 g/24 h (Bader et al. 2020; Weihrauch et al. 2000) liegt (in 8 h entsprechend 0,43 g Kreatinin; 7,2 mg Methoxyessigsäure/0,43 g Kreatinin). Die Einbeziehung des geschätzten Akkumulationsfaktors von 5 für die Extrapolation einer 8-Stunden-Exposition auf das Fließgleichgewicht (siehe Abschnitt 3.1) führt bei wiederholter Exposition gegen 5 ml 2-Methoxyethanol/m^3^ zu einer Ausscheidung von 83,5 mg Methoxyessigsäure/g Kreatinin (5 × 16,7 mg Methoxyessigsäure/g Kreatinin). Eine wiederholte Exposition gegen 1 ml 2-Methoxyethanol/m^3^ entspricht daher einer maximalen Urinkonzentration von 16,7 mg Methoxyessigsäure/g Kreatinin im Fließgleichgewicht.

Wenn diese Umrechnung für die Ableitung des MAK-Wertes von 2-Methoxyethanol aus dem BAT-Wert von 15 mg Methoxyessigsäure/g Kreatinin zugrunde gelegt wird, wird eine Luftkonzentration von 0,9 ml 2-Methoxyethanol/m^3^ ermittelt.

Daher wird der MAK-Wert von **1 ml/m^3^ (3,2 mg/m^3^)** beibehalten.

Der direkte Hautkontakt mit 2-Methoxyethanol ist zu vermeiden.

**Spitzenbegrenzung. **Da der MAK-Wert aufgrund des systemischen Effekts abgeleitet worden ist, bleibt die Zuordnung zur Kurzzeitwert-Kategorie II bestehen. Wegen der langen Halbwertszeit des kritischen Metaboliten Methoxyessigsäure erfolgt die Begrenzung von Expositionsspitzen weiterhin mit dem Überschreitungsfaktor 8.

**Fruchtschädigende Wirkung. **Es liegen mehrere Studien zur Untersuchung des Einflusses einer beruflichen Exposition gegen Glykolether auf das Auftreten von kongenitalen Fehlbildungen vor, die aber keine konkrete Aussage zu möglichen entwicklungstoxischen Effekten von 2-Methoxyethanol zulassen.

Die bewertungsrelevanten NOAEC/NOAEL und LOAEC/LOAEL für Entwicklungstoxizität bei Ratte, Maus, Kaninchen und Affe sind in Tabelle 4 dargestellt. Die NOAEC für Entwicklungstoxizität liegen bei der Ratte bei 10 ml 2-Methoxyethanol/m^3^ und beim Kaninchen bei 3 ml 2-Methoxyethanol/m^3^. Für orale Gabe lässt sich nur für die Ratte ein NOAEL für Entwicklungstoxizität von 16 mg/kg KG und Tag ableiten. 

Seit dem Jahr 1985 ist 2-Methoxyethanol der Schwangerschaftsgruppe B zugeordnet.

**Hinweis auf Voraussetzung für Schwangerschaftsgruppe C**

Für die Situation am Arbeitsplatz sind die Inhalationsstudien am relevantesten. Daher werden als Ausgangspunkt die NOAEC für Entwicklungstoxizität bei der Ratte von 10 ml 2-Methoxyethanol/m^3^ und beim Kaninchen von 3 ml 2-Methoxyethanol/m^3^ verwendet. Für die Extrapolation der Rattendaten auf die Situation des Menschen am Arbeitsplatz wird das PBPK-Modell von Gargas et al. (2000) zugrunde gelegt (10 ml 2-Methoxyethanol/m^3^ bei der trächtigen Ratte entsprechen 12 ml 2-Methoxyethanol/m^3^ bei 8-stündiger Exposition beim Menschen). Zusätzlich sind die dermale Aufnahme aus der Gasphase, die etwa so hoch ist wie die inhalative Aufnahme, und das erhöhte Atemvolumen am Arbeitsplatz zu berücksichtigen (Probandenstudie mit Maskeninhalation in Ruhe). Da bei der Umrechnung von der Tierversuchsexposition auf die Arbeitsplatzexposition im Faktor (1:2) das erhöhte Atemvolumen und die Extrapolation von 6 h aus dem Tierversuch auf 8 h am Arbeitsplatz berücksichtigt wird, das PBPK-Modell aber die Zeitextrapolation bereits enthält, ergibt sich für das erhöhte Atemvolumen ein Faktor von 0,66 (1:1,5; Hartwig und MAK Commission 2017). Daraus resultiert eine Luftkonzentration c für die inhalative und dermale Aufnahme von 4,77 ml 2-Methoxyethanol/m^3^ (c/0,66 + c = 12 ml/m^3^). Für das Kaninchen gibt es kein PBPK-Modell, daher errechnet sich aus der NOAEC von 3 ml/m^3^ unter Berücksichtigung der 6‑stündigen Exposition und des erhöhten Atemvolumens (1:2) eine Luftkonzentration von 1,5 ml 2‑Methoxyethanol/m^3^. Die dermale Aufnahme aus der Gasphase ist bereits beinhaltet, da die Tiere ganzkörperexponiert worden sind. Zudem wird angenommen, dass die dermale Aufnahme bei Kaninchen und Mensch gleich hoch ist. Ausgehend von der niedrigeren der beiden errechneten Luftkonzentrationen von 1,5 ml/m^3^ ergibt sich, dass bis zu einer Luftkonzentration von **0,15 ml 2-Methoxyethanol/m^3^** eine fruchtschädigende Wirkung nicht anzunehmen ist.

**Hautresorption. **2-Methoxyethanol ist mit „H“ markiert (Hartwig 2009; Henschler 1983). Der MAK-Wert hat sich durch die Reevaluation nicht geändert, so dass die Markierung mit „H“ weiterhin bestehen bleibt.

**Sensibilisierende Wirkung. **Ein bereits im letzten Nachtrag von 2009 (Hartwig 2009) aufgeführter Maximierungstest an Meerschweinchen weist nicht auf eine kontaktsensibilisierende Wirkung hin. Auch aufgrund der Molekülstruktur besteht kein Verdacht auf eine sensibilisierende Wirkung. Daten zur atemwegssensibilisierenden Wirkung fehlen. Die Substanz wird daher weiterhin weder mit „Sa“ noch mit „Sh“ markiert.
